# The role of the first interpersonal trauma exposure’s developmental period on fear regulation processes among adult women

**DOI:** 10.1080/20008066.2025.2587483

**Published:** 2025-11-19

**Authors:** Emilie Rudd, Christine Truong, Alexe Bilodeau-Houle, Myriam Beaudin, Valérie Bouchard, Marie-France Marin

**Affiliations:** a Department of Psychology, Université du Québec à Montréal, Montréal, Canada; b School of Criminology, Université de Montréal, Montréal, Canada; c Department of Psychology, Université du Québec à Montréal, Montreal Mental Health University Institute Research Center, Montréal, Canada

**Keywords:** Interpersonal trauma, post-traumatic stress disorder, women, fear extinction learning, fear regulation, skin conductance responses, developmental periods, Trauma interpersonal, trastorno de estrés postraumático, mujeres, aprendizaje de extinción del miedo, regulación del miedo, respuesta de conductancia de la piel, periodos del desarrollo

## Abstract

**Background:** Interpersonal trauma is associated with a higher risk of developing symptoms of post-traumatic stress disorder (PTSD) following trauma exposure. PTSD, which is more prevalent among women, is characterised by heightened fear and difficulties regulating it. Although fear regulation deficits in PTSD are well documented, considerable variability exists in how individuals learn and regulate fear. Brain regions involved in fear learning and regulation follow distinct developmental trajectories and are more sensitive to stress at certain timepoints. As exposure to severe stress (e.g. trauma) could influence the development and/or functioning of brain regions involved in fear learning and regulation, the timing of such exposure may potentially induce differential effects.

**Objective:** This study explores the association between the developmental period – childhood (0–11 years), adolescence (12–17 years), adulthood (18 years and older) – during which the first interpersonal trauma occurred, and fear learning and regulation processes in a sample of adult women.

**Methods:** Ninety-five women with a history of interpersonal trauma reported their age at first exposure and underwent a validated two-day fear conditioning and extinction protocol (conditioning and extinction on one day, followed by extinction memory recall 24 h later). Skin conductance responses (SCR) were used to index physiological fear levels.

**Results:** During fear conditioning and extinction, no group differences emerged. During the early phase of extinction memory recall, women whose first trauma occurred during adolescence or adulthood showed higher SCRs than those exposed during childhood (Time × Trauma age group: *F*(6, 2078.05) = 7.78, *p* < .001).

**Conclusion:** These findings suggest that the developmental timing of trauma exposure influences fear regulation in adulthood, highlighting potential windows of vulnerability that could inform targeted interventions.

## HIGHLIGHTS

- The developmental timing of trauma exposure influences how extinction memories are retrieved.
- Women first exposed to trauma in childhood show lower electrodermal responses compared to those first exposed in adolescence or in adulthood in the early phase of extinction memory recall.
- These findings suggest that interventions tailored to the developmental timing of trauma exposure could enhance treatment outcomes.

## Abstract (es)

**Antecedentes:** El trauma interpersonal se asocia con un mayor riesgo de desarrollar síntomas de trastorno de estrés postraumático (TEPT) tras la exposición al trauma. El TEPT, más frecuente en mujeres, se caracteriza por un miedo intenso y dificultades para regularlo. Si bien los déficits en la regulación del miedo en el TEPT están bien documentados, existe una variabilidad importante en la forma en que las personas aprenden y regulan el miedo. Las regiones cerebrales involucradas en el aprendizaje y la regulación del miedo siguen trayectorias de desarrollo distintivas y son más sensibles al estrés en determinados momentos. Dado que la exposición a un estrés grave (ej., un trauma) podría influir en el desarrollo y/o funcionamiento de las regiones cerebrales implicadas en el aprendizaje y regulación del miedo, el momento de dicha exposición podría inducir efectos diferenciales.

**Objetivo:** Este estudio explora la asociación entre el periodo de desarrollo -infancia (0–11 años), adolescencia (12–17 años), adultez (18 años y más)- durante el cual ocurrió el primer trauma interpersonal y los procesos de aprendizaje y regulación del miedo en una muestra de mujeres adultas.

**Métodos:** Noventa y cinco mujeres con antecedentes de trauma interpersonal informaron su edad al momento de la primera exposición y se sometieron a un protocolo validado de condicionamiento y extinción del miedo de dos días (condicionamiento y extinción en un día, seguido de recuperación del recuerdo de extinción 24 horas después). Se utilizaron respuestas de conductancia de la piel (SCR por sus siglas en ingles) para medir los niveles fisiológicos del miedo.

**Resultados:** Durante el condicionamiento y la extinción del miedo, no surgieron diferencias entre los grupos. Durante la fase inicial de la recuperación del recuerdo de extinción, las mujeres cuyo primer trauma ocurrió durante la adolescencia o adultez mostraron SCRs más altas que aquellas expuestas durante la infancia (Tiempo × grupo de edad del trauma: F(6, 2078.05) = 7.78, *p* < .001).

**Conclusión:** Estos hallazgos sugieren que el momento del desarrollo en el que se produce la exposición al trauma influye en la regulación del miedo en la adultez, lo que pone de relieve posibles periodos de vulnerabilidad que podrían orientar intervenciones específicas.

## 1. Introduction

Approximately 75% of Canadians experience at least one traumatic event in their lifetime (Van Ameringen et al., 2008). While many show post-traumatic stress symptoms (PTSS) such as intrusive memories, hypervigilance, avoidance, and heightened physiological arousal, only 9% will meet the clinical criteria for a diagnosis of post-traumatic stress disorder (PTSD) (Breslau, 2009; Van Ameringen et al., 2008). Many individuals exhibit some symptoms without meeting the full diagnostic criteria, underscoring the importance of considering a dimensional approach when studying trauma exposure and PTSD. This disorder is also characterised by high comorbidity rates, particularly with depression, anxiety, and substance use disorders, which exacerbate physiological distress and impair social and occupational functioning (Kessler, 1995; Ozer et al., 2003). These statistics highlight the need to identify factors that predispose certain individuals to develop PTSS and PTSD.

One such factor is sex, as women are twice as likely as men to develop PTSD (Breslau, 2009; Kessler, 1995; Kilpatrick et al., 2013), and are more prone to severe and chronic PTSS (Kilpatrick et al., 2013; Kimerling et al., 2018; Tolin & Foa, 2008). Indeed, PTSS tend to persist up to four times longer in women than in men (Blain et al., 2010; Kimerling et al., 2018; Tolin & Foa, 2008). Despite this vulnerability, women remain underrepresented in PTSS and PTSD research (Haering et al., 2024). Although men experience more traumatic events overall (Tolin & Foa, 2008), women are disproportionately exposed to sexual assaults and rape, forms of interpersonal trauma. Interpersonal traumas are defined as events involving intentional harm from others (Breslau, 1991; Kessler, 1995; Lilly & Valdez, 2012) and they are particularly harmful, leading to more severe outcomes and a higher risk of developing PTSS than non-interpersonal traumas (Chapman et al., 2004; Forbes et al., 2010; Kessler, 1995; Kessler et al., 2005). Even when trauma type is controlled, women remain more vulnerable, likely due to biological, psychological, and social risk factors (Blain et al., 2010; Kimerling et al., 2018; Lebron-Milad & Milad, 2012; Olff et al., 2007; Pineles et al., 2017).

Beyond sex-related vulnerability and trauma type, trauma chronicity also plays a critical role. Repeated or sustained interpersonal trauma, such as chronic abuse, incest, or prolonged domestic violence, are particularly detrimental, as they increase the likelihood of developing complex posttraumatic stress disorder (CPTSD) (Giourou et al., 2018). Unlike PTSD and PTSS which are characterised by an exacerbated fear response and difficulty regulating it (Blechert et al., 2007), CPTSD encompasses additional symptom domains including pervasive emotional dysregulation, negative self-concept, and relational disturbances, reflecting the profound impact of early, chronic trauma (Briere & Elliott, 2003; Cloitre et al., 2009, 2013; McLaughlin et al., 2014).

Because fear regulation represents a core mechanism underlying both PTSD and PTSS, it has been the focus of extensive research. Clinically, impaired fear regulation manifests through intrusive symptoms that trigger intense fear and physiological arousal, hyperarousal symptoms such as hypervigilance and exaggerated startle responses, and persistent difficulties downregulating fear once activated. Although avoidance behaviours are intended to reduce the activation of fear responses, they paradoxically perpetuate difficulties in regulating fear responses. To study fear regulation, researchers use validated fear conditioning and extinction protocols (Garfinkel et al., 2014; Helpman et al., 2016; Milad & Quirk, 2012; Rougemont-Bücking et al., 2011). Importantly, these protocols are not limited to the study of PTSD and PTSS. They are also useful for examining fear regulation processes in other psychopathologies, such as anxiety disorders, and to study such processes among general populations. In these protocols, a neutral stimulus (e.g. coloured lamp) is paired with an aversive unconditioned stimulus (US; e.g. electric shock) and becomes a conditioned stimulus (CS+) that elicits fear responses. This conditioning phase is followed by an extinction phase, where the CS+ is presented repeatedly without the US, reducing fear responses. After a delay (usually 24 h), extinction memory recall is tested by re-exposing participants to the CS+ without the US. Low fear levels suggest successful consolidation and recall of the safety memory formed during extinction. Physiological measures such as skin conductance responses (SCRs) are commonly used to quantify fear responses (Drexler et al., 2018; Marin et al., 2016; Stockhorst & Antov, 2016). There are several advantages to using SCRs as this measure is non-invasive, relatively inexpensive compared to other methods (e.g. fMRI), easy to use, and highly sensitive to temporal variations, allowing to capture subtle changes in physiological activation in response to stimuli. These methodological advantages explain its widespread use in fear conditioning and extinction protocols.

Research comparing trauma-exposed and control groups have shown mixed results for conditioning and extinction (Blechert et al., 2007; Glover et al., 2011), but more consistent differences during the extinction memory recall (Garfinkel et al., 2014; Helpman et al., 2016; Marin et al., 2016; Milad et al., 2008; Wicking et al., 2016). Individuals with PTSD often exhibit elevated SCRs to the CS+ in this phase compared to trauma-exposed controls without PTSD, suggesting difficulty retrieving the safety memory formed during extinction (Garfinkel et al., 2014; Helpman et al., 2016; Marin et al., 2016; Milad et al., 2008; Wicking et al., 2016). Findings indicate that deficits in extinction memory recall, rather than in conditioning and extinction learning, may be a core feature of PTSD.

Fear learning and regulation rely on coordinated activity of the amygdala, hippocampus, and prefrontal cortex (Shin & Liberzon, 2010). The amygdala detects threats and activates fear responses (Adolphs et al., 1995; LeDoux, 2000), the hippocampus encodes context and differentiates safe from threatening environments (Milad et al., 2007), and the prefrontal cortex exerts top-down regulation to inhibit fear responses (Shin & Liberzon, 2010). Consistent with behavioural findings, trauma-related alterations have been observed in the neuronal circuits underlying fear processing (Stevens et al., 2013). In PTSD, amygdala hyperactivation heightens threat detection, hippocampal hypoactivation disrupts contextual memory (Garfinkel et al., 2014; Milad et al., 2009; Rougemont-Bücking et al., 2011), and prefrontal cortex hypoactivation impairs fear regulation (Fonzo et al., 2017; Hopper et al., 2007). These patterns reflect alterations of the fear circuitry, which may underlie the deficits observed in extinction memory recall.

Developmental trajectories differ among these regions: the hippocampus matures early in childhood, the amygdala develops through adolescence into adulthood, and the prefrontal cortex reaches maturity by late adolescence or early adulthood (Lupien et al., 2009; Uematsu et al., 2012). Childhood (0–11 years) features high plasticity in limbic regions such as the amygdala and hippocampus, increasing vulnerability to stress effects on emotional reactivity and threat processing (Pechtel & Pizzagalli, 2011; Tottenham & Sheridan, 2010). Adolescence (12–17 years) involves prefrontal remodelling, making it sensitive to regulatory disruptions (Lupien et al., 2009; McLaughlin et al., 2014). By adulthood (18 years and older), most fear-related regions are mature. Thus, childhood and adolescence may represent sensitive periods for fear regulation development. Consequently, exposure to major stressors like traumatic events during these periods can influence the maturation of fear-related circuits, thereby increasing long-term psychological risk (Andersen et al., 2008; Pechtel & Pizzagalli, 2011). This aligns with the Life Cycle Model of Stress, which states that trauma effects are shaped not only by its severity or chronicity, but also by the developmental period at which the exposure occurs, reflecting neural vulnerability across the lifespan (Lupien et al., 2009). According to this model, the period of highest vulnerability to stress are early childhood for hippocampus, and childhood through early adulthood for the amygdala and prefrontal cortex.

Although neurobiological mechanisms underlying PTSS have been studied, interindividual differences remain poorly understood. The developmental timing of first trauma exposure, particularly interpersonal trauma, is an underexplored factor that may influence fear regulation circuits and PTSS risk. Impaired fear regulation contributes to both PTSS onset and persistence (Morina et al., 2014; North et al., 2012). Addressing this gap is important for individuals exposed to interpersonal violence, as they often exhibit persistent fear-related symptoms (Morina et al., 2014).

This study examines the association between the developmental period of first interpersonal trauma exposure (i.e. childhood, adolescence, or adulthood) and fear learning and regulation in adult women. To reduce heterogeneity and better isolate developmental timing effects, we focus exclusively on women, who are more vulnerable to PTSD. We hypothesise that trauma timing will be associated with distinct patterns of fear regulation, with the strongest differences emerging during extinction memory recall. Specifically, women first exposed in childhood or adolescence are expected to show higher fear responses at extinction memory recall compared to those exposed in adulthood, reflecting the varying developmental sensitivity of fear-related brain regions.

## 2. Methods

### 2.1. Participants

Data were drawn from a larger study investigating the intergenerational transmission of vulnerability and resilience mechanisms in children of mothers with a history of interpersonal trauma. A total of 121 adult women who had experienced interpersonal trauma were recruited through advertisements in local stores, trauma clinics, community organisations, and on social media (e.g. Facebook). Eligibility was determined through a telephone screening. Exclusion criteria were: (i) inability to understand and speak French or English; (ii) abnormal or uncorrected vision; (iii) history of brain damage; (iv) chronic, unstable, or severe medical conditions; (v) pregnancy; (vi) current diagnosis of psychotic or bipolar disorder; (vii) multiple trauma exposures without identifying interpersonal trauma as the most significant. This study was approved by the ethics committee of the Centre intégré universitaire de santé et de services sociaux de l'Est-de-l'Île-de-Montréal. All participants signed a consent form in accordance with the Declaration of Helsinki and received $115 in compensation.

### 2.2. Questionnaires

#### 2.2.1. Demographics

Participants completed a demographic questionnaire assessing family composition, ethnicity and highest education level completed.

#### 2.2.2. Life-events checklist for DSM-5 (LEC-5)

Trauma exposure across the lifespan was assessed using a French version of the LEC-5 (double-blind back translation by our laboratory), which includes 17 items describing stressful events that are known to potentially result in PTSD (Stevens et al., 2018). For each item, participants indicated whether the event: (a) happened to them; (b) was witnessed; (c) happened to someone close; (d) occurred in a work context; (e) was uncertain. The LEC-5 shows good test-retest reliability (*r* = 82) and sufficient inter-rater reliability (κ = .61) (Gray et al., 2004). To determine if these events met DSM-5 Criterion A for PTSD (American Psychiatric Association, 2013), participants indicated, as supplementary components, whether their life or physical integrity was threatened or if they were seriously injured. A trauma exposure score was calculated by summing events identified as traumatic, as opposed to stressful. As an additional measure, participants reported the age at which each event occurred, allowing to determine the developmental period of first interpersonal trauma exposure. Based on neurodevelopmental models of limbic and prefrontal regions maturation (Blakemore, 2012; Jaworska & MacQueen, 2015) and puberty onset (around 12 years old) (Farello et al., 2019), developmental periods were categorised as childhood (0–11 years), adolescence (12–17 years), and adulthood (18 years and older). Additionally, a trauma recency score was computed by subtracting age at most recent trauma from the participant’s current age to measure of how recent the last trauma was. Interpersonal trauma subtypes was coded into six categories: (1) repeated sexual, (2) non-repeated sexual abuse, (3) repeated physical abuse, (4) non-repeated physical abuse, (5) repeated organised violence, and (6) non-repeated organised violence. ‘Repeated’ was operationalised as the same trauma occurring on more than one occasion, based on the participant’s self-report whereas ‘non-repeated’ indicated a single occurrence.

#### 2.2.3. PTSD checklist for DSM-5 (PCL-5)

PTSS clusters were assessed using a French version of the PCL-5 (Ashbaugh et al., 2016; Weathers et al., 2013), a 20-item self-report questionnaire evaluating symptoms over the past month on a 5-point Likert scale. Scores range from 0 to 80, with higher scores reflecting greater PTSS severity. The original PCL-5 and French versions demonstrate excellent internal consistency (α = .94) and strong test-retest reliability (*r* = .82 and .89 respectively) (Ashbaugh et al., 2016). In our sample, the PCL-5 also shows excellent internal consistency (α = .92).

#### 2.2.4. State-trait anxiety inventory (STAI-T)

Given that anxiety frequently co-occurs with PTSD (van Minnen et al., 2015) and may influence psychological and physiological fear-related processes (Lonsdorf & Merz, 2017), trait anxiety was assessed using the trait scale of a French-Canadian revised STAI-T (Gauthier & Bouchard, 1993; Spielberger, 1983). Participants rated 20 items on a 4-point Likert scale. Scores range from 20 to 80, with higher scores indicating greater trait anxiety. The original trait subscale demonstrates excellent internal consistency (α = .90) and test-retest reliability (*r* = .86) (Lonsdorf & Merz, 2017), matched by the French-Canadian adaptation (α = .90) (Gauthier & Bouchard, 1993). In our sample, the STAI-T also exhibits excellent internal consistency (α = .92).

#### 2.2.5. Beck depression inventory-II (BDI-II)

Since depression is a common comorbidity of PTSD (Rytwinski et al., 2013), depressive symptoms were assessed using a French revised BDI-II (Beck et al., 1988; Bourque & Beaudette, 1982). This 21-item questionnaire evaluates depression symptoms over the past two weeks. Each item is rated from 0 to 3, yielding total scores from 0 to 63, with higher scores indicating greater symptom severity. The original version typically shows excellent internal consistency, with an average alpha coefficient around 0.9 (range 0.83–0.96) (Wang & Gorenstein, 2013), and the French version demonstrates similar reliability (α = .92) (Bourque & Beaudette, 1982). In our sample, the BDI-II also demonstrates excellent internal consistency (α = .93).

### 2.3. Fear conditioning protocol

**Figure 1.**
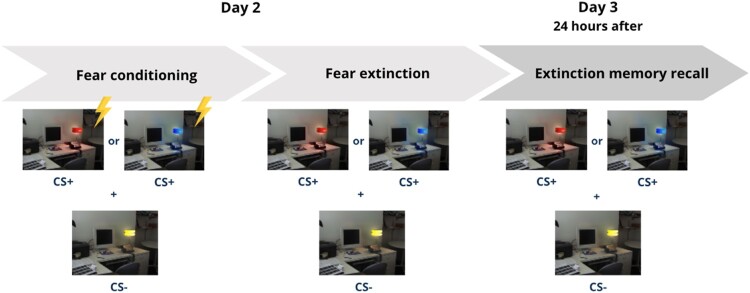
Summary of the two-day fear conditioning and extinction protocol (habituation not shown). During the conditioning phase, participants were exposed to one lamp (e.g. blue or red; conditioned stimulus, CS+), on eight occasions, five of which were paired with a 500 ms electric shock (reinforced trials). A different lamp (e.g. yellow; non-conditioned stimulus, CS-) was presented four times without shocks. After conditioning, participants entered the extinction phase, where both the CS+ and CS- were each presented 12 times without any shocks, allowing for the gradual extinction of the conditioned response. The next day, participants completed the extinction memory recall phase, during which the CS+ and CS- were each presented 12 times.

Participants completed a two-day fear conditioning and extinction protocol, adapted from Milad and colleagues (Milad et al., 2005, 2008, 2009) and modified into a dyadic version validated by our laboratory (Marin et al., 2020) (see Figure 1). Skin conductance responses (SCRs) were recorded using Ag/AgCl electrodes on the left palm, while stimulating electrodes were positioned on the right hand’s index and middle fingers. On the first day of the protocol, participants selected a shock level that was uncomfortable but not painful (range: 0.8−6.0 mA). They underwent the habituation phase, during which two coloured lamps (e.g. blue, red or yellow) were each presented twice without electric shocks (4 trials total). In the subsequent conditioning phase, one lamp (e.g. blue or red; conditioned stimulus, CS+) was presented eight times, with five presentations paired with a 500 ms shock (reinforced trials). The other lamp (e.g. yellow; non-conditioned stimulus, CS-) was shown four times without shocks. Following conditioning, participants entered the extinction phase, during which the CS+ and CS- were each presented 12 times without any shocks, allowing the conditioned response to extinguish.

Twenty-four hours later, participants completed the extinction memory recall phase featuring 12 presentations of both the CS+ and CS-. This phase assessed whether the safety memory formed during extinction had been consolidated and could be recalled. CS+ colours (e.g. red/blue) were counterbalanced across participants.

Across all protocol phases, each trial began with a 9–15-second black screen (intertrial interval), followed by a 3-second presentation of an office with a lamp turned off (context). Then, the lamp turned on in a specific colour for 6 s, with CS+ and CS- trials presented in an intermixed order. Reinforced trials ended with a 0.5-second shock. Unlike the original protocol, the context remained unchanged throughout the experiment.

### 2.4. General procedure

Details of the procedure are presented in Supplementary materials (see Figure S1).

### 2.5. Physiological recordings

SCRs were recorded using BIOPAC technology and AcqKnowledge software (Biopac® Systems Inc., Goleta, CA) and extracted with Matlab-based software Ledalab (www.ledalab.de). The Continuous Decomposition Analysis (CDA) decomposed SCRs into phasic responses using a 1-second post-cue latency window and a 0.01 µS amplitude threshold (Benedek & Kaernbach, 2010). SCRs values were square-root-transformed for normalisation, following standard SCRs analysis practices (Kreutzmann et al., 2021; Marin et al., 2020; Milad et al., 2009).

### 2.6. Statistical analyses

Analyses were performed in R (R Core Team, 2023; Version 4.3.2), using *lme4* (Bates et al., 2015) and *lmerTest* (Kuznetsova et al., 2017) packages.

During conditioning (eight CS+ and four CS− trials), CS+ SCRs were averaged in pairs (trials 1–2, 3–4, 5–6, and 7–8) to create four CS+ values, improving the balance and reliability of SCRs trajectories modelling across time and enabling direct comparisons between stimulus types in linear mixed-effects models. This binning strategy follows recommendations for addressing unequal trial counts (Lonsdorf et al., 2017). For the extinction learning and extinction memory recall phases (12 CS+ trials, 12 CS- trials), all SCRs were analyzed across trials without binning, as the number of CS+ and CS- trials was equivalent.

For all subsequent analyses, trauma age group refers to the three categories based on the developmental period of participants’ first exposure to interpersonal trauma: childhood, adolescence, or adulthood.

#### 2.6.1. Preliminary analyses

We examined whether trauma age group was associated with potential covariates: participants’ current age, US intensity, PCL-5 score, BDI-II score, STAI-T score, trauma exposure score, trauma recency score, and interpersonal trauma subtypes as prior literature shows that these variables may influence fear and stress responses (Epel et al., 2018; Lonsdorf et al., 2017). One-way ANOVAs tested group differences, with Tukey’s HSD post hoc tests for significant effects. Chi-square tests compared ethnicity and education across groups, as these factors can influence SCRs (Alexandra Kredlow et al., 2017).

#### 2.6.2. Main analyses

We applied a multilevel linear mixed-effects models for conditioning, extinction, and extinction memory recall. Participant ID was entered as a random intercept to account for repeated measures and within-subject dependency, given strong correlations between CS+ and CS- SCRs across phases (Barr et al., 2013; Matuschek et al., 2017): conditioning (*r* = .94, *p* < .001), extinction (*r* = .95, *p* < .001), and extinction memory recall (*r* = .96, *p* < .001),

Model selection involved comparing linear, quadratic, and cubic representations of trial effects using the Bayesian Information Criterion (BIC) (Field et al., 2012). The linear model had the poorest fit (BIC = 3200.2), the quadratic model improved fit substantially (BIC = 2885.2), and the cubic model provided the best fit (BIC = 2852.5) for all phases. After, we included the following fixed effects: stimulus type (CS+ and CS-), trauma age group, and time modelled as cubic polynomial. Model comparisons indicated that the full model including the three-way interaction (stimulus type × time × trauma age group) significantly improved model fit (χ²(12) = 83.63, *p* < .001).

Visual inspection of residual plots confirmed that the final models met the assumptions of homoscedasticity and normality. Significant interactions were decomposed using the *emmeans* package (Lenth et al., 2025) to estimate marginal means and conduct pairwise comparisons. In most cases, means were computed across time points modelled as a polynomial for each trauma age group to capture the evolution of SCRs over time. *Emmeans* was also used to examine other interaction effects not involving time (e.g. stimulus type × trauma age group). Pairwise comparisons between trauma age group were adjusted using the Tukey method.

## 3. Results

### 3.1. Preliminary analyses

Four participants were excluded due to missing data on the age of their first trauma, five for reporting a non-interpersonal trauma as the most significant event during Day 1, and 17 withdrew after Day 1 (clinical interview). The final sample included 95 trauma-exposed participants: 37 first exposed in childhood, 26 in adolescence, and 32 in adulthood (see Table 1).

**Table 1.** Participants’ demographic and clinical variables by trauma age group (i.e. developmental period of first interpersonal trauma exposure).

Variables	Trauma age group			*p*
	Childhood (*n* = 37)	Adolescence (*n* = 26)	Adulthood (*n* = 32)	
Age (years)	41.60 (4.92)	41.30 (5.19)	41.80 (4.67)	.923
PCL-5	16.10 (14.60)	12.5 (10.30)	9.90 (13.10)	.133
Trauma exposure score	4.05 (2.31)	4.42 (2.44)	4.42 (2.23)	.756
Trauma recency score (years)	21.70 (12.6)	17.10 (10.80)	11.60 (9.02)	.001
US intensity (mA)	1.76 (1.32)	1.73 (1.54)	1.53 (0.96)	.743
STAI-T	41.60 (9.16)	41.20 (10.30)	39.00 (1.60)	.581
BDI-II	10.60 (10.50)	10.00 (8.20)	7.19 (9.43)	.332
Interpersonal trauma subtypes				<.001
Repeated sexual abuse	16 (43.2%)	7 (26.9%)	2 (6.5%)	
Non-repeated sexual abuse	6 (16.2%)	14 (53.8%)	12 (38.7%)	
Repeated physical abuse	13 (35.1%)	2 (7.7%)	8 (25.8%)	
Non-repeated physical abuse	1 (2.7%)	3 (11.5%)	8 (25.8%)	
Repeated organised violence	1 (2.7%)	0 (0.0%)	1 (3.2%)	
Non-repeated organised violence	0 (0.0%)	0 (0.0%)	0 (0.0%)	
Highest education level completed				.240
Elementary school	1 (2.7%)	0 (0.0%)	0 (0.0%)	
High school	5 (13.5%)	1 (3.8%)	1 (3.1%)	
College	9 (24.3%)	6 (23.1%)	5 (15.6%)	
Bachelor	12 (32.4%)	9 (34.6%)	12 (37.5%)	
Master	8 (21.6%)	8 (30.8%)	14 (43.8%)	
Ph.D.	0 (0.0%)	2 (7.7%)	0 (0.0%)	
Postdoc	1 (2.7%)	0 (0.0%)	0 (0.0%)	
Ethnicity				.495
White	30 (81.1%)	23 (88.5%)	27 (84.4%)	
Asian	1 (2.7%)	0 (0.0%)	0 (0.0%)	
Black	0 (0.0%)	0 (0.0%)	0 (0.0%)	
Hispanic	1 (2.7%)	1 (3.8%)	3 (9.4%)	
Indigenous	0 (0.0%)	1 (3.8%)	0 (0.0%)	
Other	4 (10.8%)	1 (3.8%)	2 (9.4%)	

Note: PCL-5 = PTSD Checklist for DSM-5; US = unconditioned stimulus (shock); STAIT-T = State-Trait Anxiety Inventory; BDI-II = Beck Depression Inventory-II. Values for age, PCL-5, trauma exposure score, trauma recency score, US intensity, STAI-T, and BDI-II represent group means (standard deviations). Interpersonal trauma subtypes, ethnicity and highest education level completed are reported as N (%). For the following variables, data were missing for a small number of participants: PCL-5 (*n* = 4), trauma exposure score (*n* = 3), trauma recency score (*n* = 3), US intensity (*n* = 3), STAI-T (*n* = 3), BDI-II (*n* = 4), highest education level completed (*n* = 1), and ethnicity (*n* = 1). Analyses were conducted using available data for each variable.

No significant group differences emerged for age, highest education level completed, ethnicity, trauma exposure score, US intensity, or clinical measures (STAI-T, BDI-II, PCL-5) (*all ps* > .13). Therefore, only the models without these covariates are reported for simplicity. However, a significant difference was observed for trauma recency score, *F*(2, 91) = 7.01, *p* = .001. Participants first exposed to trauma in adulthood had a significantly shorter time since the most recent trauma than those first exposed in childhood (*p* = .001). No other group differences were significant (*all ps* > .15). Interpersonal trauma subtype distributions also differed (Fisher’s exact test, *p* *=* .0003). Participants first exposed in childhood reported more repeated sexual abuse than participants first exposed in adulthood. Participants first exposed in adolescence reported more non-repeated sexual abuse compared to participants first exposed in childhood and participants first exposed in adulthood reported more non-repeated physical abuse than those first exposed in childhood. No other group differences were significant (*all ps* > .06). Trauma recency score and interpersonal trauma subtypes were thus included as covariates in the main models, but the results remained the same with or without these covariates. Therefore, the models without covariates are reported for simplicity (see Supplementary materials Table S1 for models including these covariates).

### 3.2. Main analyses

#### 3.2.1. Conditioning

**Figure 2.**
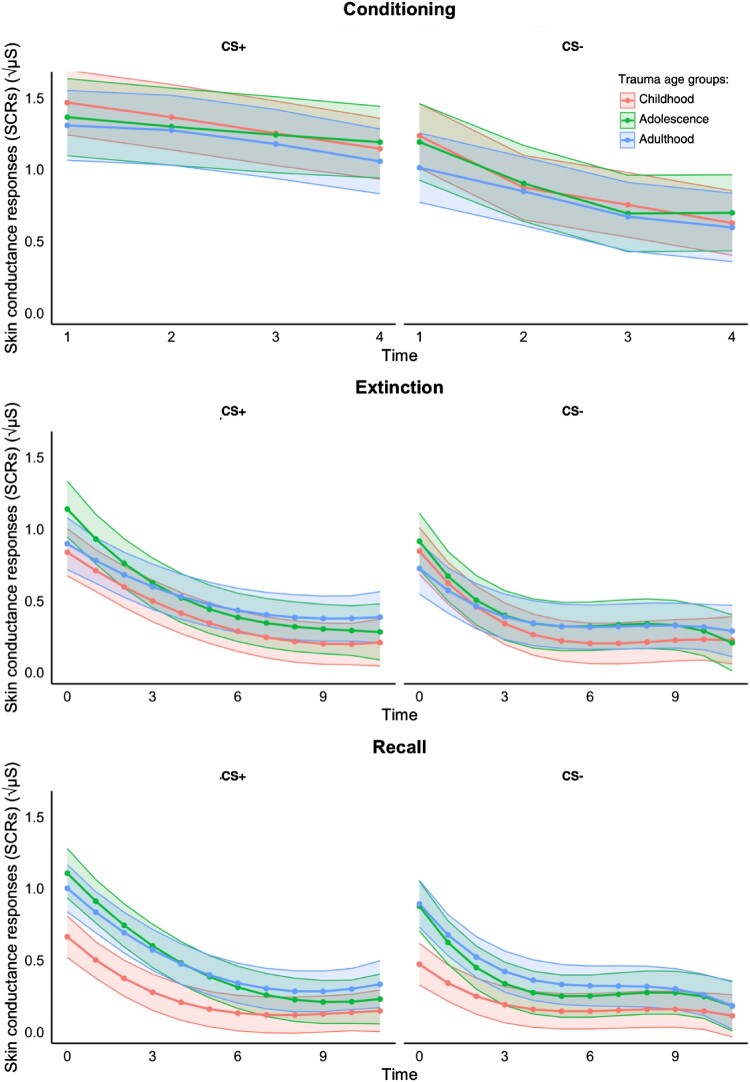
Skin conductance responses (SCRs) during conditioning, extinction, and extinction memory recall phases, as a function of trauma age group (i.e. developmental period of participants’ first exposure to interpersonal trauma), and stimulus type. SCRs were measured in microsiemens. Lines represent trauma-exposed participants, categorised by age at first interpersonal trauma: Red for childhood exposure (0–11 years), green for adolescence exposure (12–17 years), and blue for adulthood exposure (18+ years).

This model included stimulus type, time, and trauma age group (see Table 2; see Figure 2). A significant stimulus type × time interaction indicated different SCRs trajectories for CS+ and CS−. SCRs were higher for CS+ than CS− at each trial (*all ps* < .001), with differences increasing from trial 1 (*Δ* = 0.23, SE = 0.06, 95% CI [0.12, 0.35], *p* < .001) to trial 3 (*Δ* = 0.52, SE = 0.06, 95% CI [0.41, 0.63], *p* < .001) and stabilising by trial 4 (*Δ* = 0.49, SE = 0.05, 95% CI [0.39, 0.59], *p* < .001). These findings suggest a successful acquisition of conditioned responses. No other interactions were significant, though time had a main effect, reflecting a general decline in SCRs over trials.

**Table 2.** Effects for conditioning, extinction, and extinction recall models.

Effect	*F*(d*f*_1_, d*f*_2_)	*p*
*Conditioning*		
Trauma Age Group	*F*(2, 715.13) = 0.38	.69
Stimulus Type	*F*(1, 626.93) = 0.92	.34
Time	*F*(3, 626.98) = 32.46	<.001
Trauma Age Group × Stimulus Type	*F*(2, 626.92) = 0.52	.60
Trauma Age Group × Time	*F*(6, 626.98) = 0.73	.62
Stimulus Type × Time	*F*(3, 626.92) = 4.54	.004
Trauma Age Group × Stimulus Type × Time	*F*(6, 626.91) = 0.29	.94
*Extinction*		
Trauma Age Group	*F*(2, 90.83) = 0.64	.53
Stimulus Type	*F*(1, 2115.94) = 49.81	<.001
Time	*F*(3, 2116.12) = 226.40	<.001
Trauma Age Group × Stimulus Type	*F*(2, 2115.93) = 2.64	.07
Trauma Age Group × Time	*F*(6, 2116.10) = 3.56	.002
Stimulus Type × Time	*F*(3, 2115.88) = 8.70	<.001
Trauma Age Group × Stimulus Type × Time	*F*(6, 2115.88) = 0.93	.48
*Extinction memory recall*		
Trauma Age Group	*F*(2, 88.89) = 3.78	.027
Stimulus Type	*F*(1, 2077.97) = 33.01	<.001
Time	*F*(3, 2078.05) = 268.72	<.001
Trauma Age Group × Stimulus Type	*F*(2, 2077.97) = 2.25	.11
Trauma Age Group × Time	*F*(6, 2078.05) = 7.78	<.001
Stimulus Type × Time	*F*(3, 2077.99) = 14.41	<.001
Trauma Age Group × Stimulus Type × Time	*F*(6, 2077.99) = 1.34	.24

Note: *F* = F-ratio; d*f* = degrees of freedom.

The final model explained 71.9% of SCRs variance, with fixed effects accounting for 11.4%.

#### 3.2.2. Extinction

This model included stimulus type, time, and trauma age group (see Table 2; see Figure 2). A significant stimulus type × time interaction indicated that extinction differed for CS+ and CS−. The SCRs were higher for CS+ than to CS− from trial 1 (*Δ* = 0.13, SE = 0.04, 95% CI [0.04, 0.22], *p* = .003) through 7 (*Δ* = 0.09, SE = 0.02, 95% CI [0.04, 0.13], *p* < .001), with no difference thereafter (all *ps* > .05). A significant time × trauma age group interaction suggested group differences in trajectories, but pairwise comparisons were non-significant (all *ps* > .18). Finally, a stimulus type × trauma age group interaction approached significance, suggesting a potential trend toward differences in CS+ vs. CS− reactivity between groups. Post hoc comparisons showed significantly higher SCRs to the CS+ than to the CS- within each trauma age group: childhood (*Δ* = 0.10, SE = 0.035, 95% CI [0.037, 0.172], *p* = .01), adolescence (*Δ* = 0.09, SE = 0.04, 95% CI [0.04, 0.17], *p* = .03), and adulthood (*Δ* = 0.13, SE = 0.04, 95% CI [0.04, 0.17], *p* < .001). The adult trauma group showed the largest CS+ vs. CS- difference, followed by childhood and adolescence groups, suggesting a possible trend toward enhanced threat discrimination in adulthood. However, trauma age groups did not differ significantly in responses to CS+ or CS- when analyzed separately. No other interactions were significant, though stimulus type and time showed main effects, indicating overall stronger responses to CS+ than CS− and a general decline in SCRs across trials.

The final model explained 64.4% of SCRs variance, with 13.3% attributable to fixed effects alone.

#### 3.2.3. Extinction memory recall

This model included stimulus type, time, and trauma age group (see Table 2; see Figure 2). A significant stimulus type × time interaction indicated higher SCRs to CS+ than CS− from trial 1 (*Δ* = 0.18, SE = 0.04, 95% CI [0.10, 0.26], *p* < .001) through 6 (*Δ* = 0.07, SE = 0.02, 95% CI [0.03, 0.11], *p* < .001), with no difference afterward (all *ps* > .05). A time × trauma age group interaction showed that participants first exposed during childhood had significantly lower SCRs, irrespective of stimulus type, than those exposed in adolescence (*ps* ≤ .009) or adulthood (*p*s ≤ .014), particularly during early trials (1–4). These differences were no longer significant by trial 6 (all *ps* > .054). No other interactions were significant, though significant main effects for stimulus type, time, and trauma age group emerged: SCRs were higher to CS+; SCRs declined across trials, and SCRs differed between groups.

The final model explained 63.8% of SCRs variance, with 18.7% attributable to fixed effects alone.

## 4. Discussion

This study examined the association between the developmental period of first interpersonal trauma exposure (trauma age group: childhood, adolescence, or adulthood) and fear learning and regulation in adult women. The most notable effect emerged during early extinction memory recall, where women first exposed to trauma in adolescence or adulthood exhibited heightened fear responses compared to those exposed in childhood. Contrary to our hypothesis, early-life trauma did not predict higher fear responses. Instead, trauma occurring later in development seemed to impair extinction memory retrieval. These findings extend prior research showing PTSD-related difficulties in extinction and recall in adulthood (Jovanovic et al., 2012; Milad et al., 2009; Pitman et al., 2012) by suggesting that trauma timing also plays a role in extinction memory retrieval. However, it is important to note that the present study relied on a non-clinical sample with predominantly subclinical PTSS. As such, the results cannot be generalised to individuals meeting diagnostic thresholds for PTSD.

Importantly, the findings cannot be fully explained by a recency effect. Although women first exposed in adulthood and those exposed in childhood differed with regards to the time since their most recent traumatic event, including this covariate in the statistical models did not alter the results. Similarly, although trauma age groups differed with regards to the interpersonal trauma subtypes, the effects remained significant when this covariate was included. Moreover, the groups did not differ significantly in terms of anxiety, depressive, or trauma-related symptoms, nor in the total number of lifetime traumatic events. These findings therefore suggest that the observed between-group differences are more likely attributable to the developmental timing of first traumatic exposure rather than to cofounding clinical or temporal factors.

These results can be better understood through the developmental neurobiology underlying fear regulation. Adolescence is a sensitive period marked by prefrontal cortex maturation and its connections with limbic structures, such as the amygdala and hippocampus (Casey et al., 2008; Tottenham & Galván, 2016). Trauma exposure during this period may interfere with these regulatory circuits, leading to long-term vulnerabilities in fear inhibition and extinction memory recall. Similarly, women first exposed to trauma in adulthood showed similar fear responses at the beginning of extinction memory recall, potentially reflecting reduced neuroplasticity after brain maturation, limiting the brain’s adaptation to traumatic experiences (Burke & Barnes, 2006; Caroni et al., 2012; Gogolla et al., 2009). PTSD has been associated with structural and functional brain changes affecting fear regulation (Bremner et al., 1995; Shin et al., 2006), including reduced synaptic plasticity, diminished neurogenesis, and altered connectivity, ultimately limiting adaptive stress responses (Krystal & Duman, 2004; Pitman et al., 2012). Our results suggest that the underlying neurodevelopmental alterations do not necessarily impair initial extinction learning but may be specific to the extinction memory recall. This aligns with prior PTSD research showing impairments during that phase (Garfinkel et al., 2014; Helpman et al., 2016; Marin et al., 2016; Milad et al., 2006, 2008, 2009; Wicking et al., 2016). One possible explanation is that extinction and extinction memory recall involve distinct neural mechanisms. While initial extinction primarily relies on short-term functional prefrontal changes, successful extinction memory recall requires consolidation and long-term retrieval of the extinction memory trace through sustained prefrontal-hippocampal interaction to contextualise the learned safety signal and inhibit the original fear memory (Milad & Quirk, 2012). Also, given that adolescence and adulthood are marked by greater cognitive, emotional, and neurobiological maturity than childhood, this could paradoxically amplify vulnerability to less adaptative fear modulation following trauma (Casey et al., 2008; Lupien et al., 2009). More developed cognitive capacities, including advanced self-awareness and autobiographical memory may potentially increase the vividness of fear-related memories (Brewin, 2011). This heightened emotional salience could therefore contribute to difficulties in extinction memory recall, resulting in higher physiological reactivity.

In contrast, women first exposed in childhood displayed lower fear responses during early extinction memory recall, suggesting more effective extinction memory retrieval. Although this result may seem counterintuitive given that childhood trauma is associated with increased risk of PTSD and other psychopathologies compared to adulthood trauma (Zlotnick et al., 2008), alternative interpretations should be considered. Early trauma is associated with early emergence of coping strategies, such as emotional avoidance, dissociation, or attentional suppression, which can reduce the intensity of emotional reactivity (McLaughlin et al., 2014; Teicher & Samson, 2016). These strategies may reflect adaptations to overwhelming stress but are not necessarily protective and may mask underlying distress (McLaughlin et al., 2014; Teicher & Samson, 2016). Learned in the context of unpredictable or threatening environments, these strategies can become ingrained and persist in adulthood (van der Kolk et al., 1996). However, women first exposed in childhood showed differentiated physiological responses during conditioning, arguing against a general blunting of emotional reactivity.

Additionally, younger children may not appraise traumatic events with the same cognitive complexity as adolescents or adults, potentially modulating the long-term impact on fear learning systems (Nelson & Gabard-Durnam, 2020). Young children have a limited understanding of abstract concepts such as causality, time, the self, and others’ intentions, which constrains their ability to contextualise an event (Flavell, 1999; Nelson & Gabard-Durnam, 2020). This limited cognitive framework may lead to trauma being encoded, remembered, and interpreted in a less elaborated and more fragmented manner. Thus, the same event may be perceived differently by an individual first exposed in childhood compared to one first exposed in adolescence, which could help explain the differences in physiological responses observed between groups. It is also possible that individuals first exposed in childhood, particularly those who are mostly asymptomatic in adulthood, may have had more developmental opportunities to differentiate between past and current threats than those first exposed in adolescence or adulthood, and therefore be better able to retain extinction memories. Future studies should assess how laboratory threats are perceived (e.g. in terms of dangerousness, painfulness, or coping efficacy) to determine whether adult-onset or adolescent-onset trauma leads to a greater level of perceived threat and, consequently, to reduced accessibility of extinction or safety memories.

From a neurodevelopmental perspective, attenuated reactivity after childhood trauma may reflect distinct neurodevelopmental adaptations during a period when limbic structures are still maturing, potentially accelerating fear circuits maturation (Pechtel & Pizzagalli, 2011; Sheridan & McLaughlin, 2014). Animal models show that early adversity can accelerate fear circuits maturation, leading to a faster transition toward habituation and avoidance behaviours in response to threatening stimuli compared to non-stressed peers (Sheridan & McLaughlin, 2014). Neuroimaging studies in humans also show that children exposed to early deprivation (e.g. institutionalisation) exhibit accelerated maturation of amygdala and medial prefrontal cortex connectivity, shifting from child-like positive coupling to adult-like negative coupling earlier in life (Gee et al., 2013). This accelerated development can reduce short-term anxiety but often coexists with structural changes like reduced prefrontal volume (Hanson et al., 2012), which are not optimal for maturation of emotion regulation systems. These interpretations align with the Stress Acceleration Hypothesis (Callaghan & Tottenham, 2016), which proposes that early-life stress can accelerate emotional development as an adaptive response to threat by prioritising the rapid maturation of fear-related circuits to enhance survival in a threatening environment. This early shift may help children respond more independently and efficiently to danger by reducing fear reactivity. However, it may also increase the risk of later psychopathology, therefore it is important not to interpret this pattern as being necessarily protective. This neurodevelopmental trajectory, combined with the emergence of particular coping strategies, may contribute to the lower fear responses observed in adult women exposed to trauma during their childhood. Future studies could examine whether these physiological patterns also reflect in neural correlates (e.g. through neuroimaging) or in the adoption of specific coping strategies.

Another possibility is that extinction memory recall was preserved because the prefrontal cortex, a key region for extinction recall (Milad et al., 2007), was not yet fully engaged in its maturation during childhood (Lupien et al., 2009; Uematsu et al., 2012). Trauma exposure during this stage may therefore have had less disruptive effects on prefrontal development compared to exposure during adolescence, a critical period of prefrontal maturation. Thus, women first exposed in childhood may have developed a prefrontal cortex along a relatively typical trajectory, which could partly explain the lower fear responses observed during recall.

### 4.1. Strengths, limitations and future directions

Although all participants experienced interpersonal trauma, most showed subclinical symptoms, which can both be a strength and a limitation. Studying a non-clinical population enables detection of subtle effects of trauma timing that might otherwise be masked by clinical severity. These findings highlight the relevance of fear learning protocols in capturing individual differences that may not be detected through diagnostic categories alone, thereby contributing to understanding trauma-related vulnerability outside of traditional diagnostic boundaries. However, the absence of PTSD diagnoses limits generalizability to clinical populations. Future studies should replicate these findings in clinically diagnosed PTSD samples, but also with a non-clinical population similar to ours since this is the first study to examine these effects.

Additional limitations include the cross-sectional design, which prevents causal inferences about developmental trajectories. Also, the use of retrospective self-report of trauma timing is subject to recall bias (Brewin, 2011). Our sample was limited to women exposed to interpersonal trauma; therefore, results may not generalise to men or to other trauma types.

Finally, we were unable to capture detailed characteristics of trauma exposure such as emotional load, perceived intensity, duration or chronicity (e.g. length of abuse, spacing of events). Although we included a trauma exposure score and distinguished between repeated and non-repeated exposures, which provided a proxy of repetition load, these measures remain limited. Future studies should incorporate richer ‘load’ indices (number × duration × chronicity × subtype) to better evaluate whether they moderate or mediate the observed effects. Future studies should also use multimodal assessments, such as neuroimaging or subjective fear reports, to capture a more global portrait of fear regulation alterations across trauma timing.

## 5. Conclusion

Our findings suggest that the timing of interpersonal trauma exposure is a relevant factor in understanding individual differences in fear regulation and highlight the importance of developmental context in assessing long-term effects of interpersonal trauma. Interventions tailored to the developmental timing of trauma could be beneficial. For individuals first exposed in adolescence or adulthood, strategies could target regulation and cognitive flexibility, whereas for childhood exposure, approaches should focus on replacing avoidance or dissociative coping strategies with more adaptive mechanisms.

## Supplementary Material

Supplementary_Material.docx

## Data Availability

The data that support the finding of this study are available on request from the corresponding author.

## References

[CIT0001] Adolphs, R., Tranel, D., Damasio, H., & Damasio, A. R. (1995). Fear and the human amygdala. *The Journal of Neuroscience*, *15*(9), 5879–5891. 10.1523/JNEUROSCI.15-09-05879.1995 7666173 PMC6577662

[CIT0002] Alexandra Kredlow, M., Pineles, S. L., Inslicht, S. S., Marin, M., Milad, M. R., Otto, M. W., & Orr, S. P. (2017). Assessment of skin conductance in African American and Non-African American participants in studies of conditioned fear. *Psychophysiology*, *54*(11), 1741–1754. 10.1111/psyp.12909 28675471 PMC5638680

[CIT0003] American Psychiatric Association. (2013). *Diagnostic and statistical manual of mental disorders* (5th ed.).

[CIT0004] Andersen, S. L., Tomada, A., Vincow, E. S., Valente, E., Polcari, A., & Teicher, M. H. (2008). Preliminary evidence for sensitive periods in the effect of childhood sexual abuse on regional brain development. *The Journal of Neuropsychiatry and Clinical Neurosciences*, *20*(3), 292–301. 10.1176/jnp.2008.20.3.292 18806232 PMC4270804

[CIT0005] Ashbaugh, A. R., Houle-Johnson, S., Herbert, C., El-Hage, W., & Brunet, A. (2016). Psychometric validation of the English and French versions of the posttraumatic stress disorder checklist for DSM-5 (PCL-5). *PLoS One*, *11*(10), e0161645. 10.1371/journal.pone.0161645 27723815 PMC5056703

[CIT0006] Barr, D. J., Levy, R., Scheepers, C., & Tily, H. J. (2013). Random effects structure for confirmatory hypothesis testing: Keep it maximal. *Journal of Memory and Language*, *68*(3), 255–278. 10.1016/j.jml.2012.11.001 PMC3881361 24403724

[CIT0007] Bates, D., Mächler, M., Bolker, B., & Walker, S. (2015). Fitting linear mixed-effects models using lme4. *Journal of Statistical Software*, *67*(1), 1–48. 10.18637/jss.v067.i01

[CIT0008] Beck, A. T., Epstein, N., Brown, G., & Steer, R. A. (1988). An inventory for measuring clinical anxiety: Psychometric properties. *Journal of Consulting and Clinical Psychology*, *56*(6), 893–897. 10.1037/0022-006X.56.6.893 3204199

[CIT0009] Benedek, M., & Kaernbach, C. (2010). Decomposition of skin conductance data by means of nonnegative deconvolution. *Psychophysiology*, *47*(4), 647–658. 20230512 10.1111/j.1469-8986.2009.00972.x PMC2904901

[CIT0010] Blain, L. M., Galovski, T. E., & Robinson, T. (2010). Gender differences in recovery from posttraumatic stress disorder: A critical review. *Aggression and Violent Behavior*, *15*(6), 463–474. 10.1016/j.avb.2010.09.001

[CIT0011] Blakemore, S.-J. (2012). Imaging brain development: The adolescent brain. *NeuroImage*, *61*(2), 397–406. 10.1016/j.neuroimage.2011.11.080 22178817

[CIT0012] Blechert, J., Michael, T., Vriends, N., Margraf, J., & Wilhelm, F. H. (2007). Fear conditioning in posttraumatic stress disorder: Evidence for delayed extinction of autonomic, experiential, and behavioural responses. *Behaviour Research and Therapy*, *45*(9), 2019–2033. 10.1016/j.brat.2007.02.012 17442266

[CIT0013] Bourque, P., & Beaudette, D. (1982). Étude psychometrique du questionnaire de dépression de Beck auprès d’un échantillon d’étudiants universitaires francophones [Psychometric study of the Beck Depression Inventory on a sample of French-speaking university students]. *Canadian Journal of Behavioural Science/Revue canadienne des sciences du comporteme*, *14*(3), 211–218.

[CIT0014] Bremner, J. D., Randall, P., Scott, T. M., Bronen, R. A., Seibyl, J. P., Southwick, S. M., Delaney, R. C., McCarthy, G., Charney, D. S., & Innis, R. B. (1995). MRI-based measurement of hippocampal volume in patients with combat-related posttraumatic stress disorder. *American Journal of Psychiatry*, *152*(7), 973–981. 10.1176/ajp.152.7.973 7793467 PMC3233767

[CIT0015] Breslau, N. (1991). Traumatic events and posttraumatic stress disorder in an urban population of young adults. *Archives of General Psychiatry*, *48*(3), 216–222. 10.1001/archpsyc.1991.01810270028003 1996917

[CIT0016] Breslau, N. (2009). The epidemiology of trauma, PTSD, and other posttrauma disorders. *Trauma, Violence, & Abuse*, *10*(3), 198–210. 10.1177/1524838009334448 19406860

[CIT0017] Brewin, C. R. (2011). The nature and significance of memory disturbance in posttraumatic stress disorder. *Annual Review of Clinical Psychology*, *7*(1), 203–227. 10.1146/annurev-clinpsy-032210-104544 21219190

[CIT0018] Briere, J., & Elliott, D. M. (2003). Prevalence and psychological sequelae of self-reported childhood physical and sexual abuse in a general population sample of men and women. *Child Abuse & Neglect*, *27*(10), 1205–1222. 10.1016/j.chiabu.2003.09.008 14602100

[CIT0019] Burke, S. N., & Barnes, C. A. (2006). Neural plasticity in the ageing brain. *Nature Reviews Neuroscience*, *7*(1), 30–40. 10.1038/nrn1809 16371948

[CIT0020] Callaghan, B. L., & Tottenham, N. (2016). The neuro-environmental loop of plasticity: A cross-species analysis of parental effects on emotion circuitry development following typical and adverse caregiving. *Neuropsychopharmacology*, *41*(1), 163–176. 10.1038/npp.2015.204 26194419 PMC4677125

[CIT0021] Caroni, P., Donato, F., & Muller, D. (2012). Structural plasticity upon learning: Regulation and functions. *Nature Reviews Neuroscience*, *13*(7), 478–490. 10.1038/nrn3258 22714019

[CIT0022] Casey, B. J., Jones, R. M., & Hare, T. A. (2008). The adolescent brain. *Annals of the New York Academy of Sciences*, *1124*(1), 111–126. 10.1196/annals.1440.010 18400927 PMC2475802

[CIT0023] Chapman, D. P., Whitfield, C. L., Felitti, V. J., Dube, S. R., Edwards, V. J., & Anda, R. F. (2004). Adverse childhood experiences and the risk of depressive disorders in adulthood. *Journal of Affective Disorders*, *82*(2), 217–225. 10.1016/j.jad.2003.12.013 15488250

[CIT0024] Cloitre, M., Garvert, D. W., Brewin, C. R., Bryant, R. A., & Maercker, A. (2013). Evidence for proposed ICD-11 PTSD and complex PTSD: A latent profile analysis. *European Journal of Psychotraumatology*, *4*(1), 20706. 10.3402/ejpt.v4i0.20706 PMC3656217 23687563

[CIT0025] Cloitre, M., Stolbach, B. C., Herman, J. L., Kolk, B. v. d., Pynoos, R., Wang, J., & Petkova, E. (2009). A developmental approach to complex PTSD: Childhood and adult cumulative trauma as predictors of symptom complexity. *Journal of Traumatic Stress*, *22*(5), 399–408. 10.1002/jts.20444 19795402

[CIT0026] Drexler, S. M., Merz, C. J., & Wolf, O. T. (2018). Preextinction stress prevents context-related renewal of fear. *Behavior Therapy*, *49*(6), 1008–1019. 10.1016/j.beth.2018.03.001 30316481

[CIT0027] Epel, E. S., Crosswell, A. D., Mayer, S. E., Prather, A. A., Slavich, G. M., Puterman, E., & Mendes, W. B. (2018). More than a feeling: A unified view of stress measurement for population science. *Frontiers in Neuroendocrinology*, *49*, 146–169. 10.1016/j.yfrne.2018.03.001 29551356 PMC6345505

[CIT0028] Farello, G., Altieri, C., Cutini, M., Pozzobon, G., & Verrotti, A. (2019). Review of the literature on current changes in the timing of pubertal development and the incomplete forms of early puberty. *Frontiers in Pediatrics*, *7*, 147. 10.3389/fped.2019.00147 31139600 PMC6519308

[CIT0029] Field, A., Miles, J., & Field, Z. (2012). *Discovering statistics using R*, 1–992.

[CIT0030] Flavell, J. H. (1999). Cognitive development: Children’s knowledge about the mind. *Annual Review of Psychology*, *50*(1), 21–45. 10.1146/annurev.psych.50.1.21 10074674

[CIT0031] Fonzo, G. A., Goodkind, M. S., Oathes, D. J., Zaiko, Y. V., Harvey, M., Peng, K. K., Weiss, M. E., Thompson, A. L., Zack, S. E., Lindley, S. E., Arnow, B. A., Jo, B., Gross, J. J., Rothbaum, B. O., & Etkin, A. (2017). Ptsd psychotherapy outcome predicted by brain activation during emotional reactivity and regulation. *American Journal of Psychiatry*, *174*(12), 1163–1174. 10.1176/appi.ajp.2017.16091072 28715908 PMC5711543

[CIT0032] Forbes, D., Creamer, M., Bisson, J. I., Cohen, J. A., Crow, B. E., Foa, E. B., Friedman, M. J., Keane, T. M., Kudler, H. S., & Ursano, R. J. (2010). A guide to guidelines for the treatment of PTSD and related conditions. *Journal of Traumatic Stress*, *23*(5), 537–552. 10.1002/jts.20565 20839310

[CIT0033] Garfinkel, S. N., Abelson, J. L., King, A. P., Sripada, R. K., Wang, X., Gaines, L. M., & Liberzon, I. (2014). Impaired contextual modulation of memories in PTSD: An fMRI and psychophysiological study of extinction retention and fear renewal. *The Journal of Neuroscience*, *34*(40), 13435–13443. 10.1523/JNEUROSCI.4287-13.2014 25274821 PMC4262698

[CIT0034] Gauthier, J., & Bouchard, S. (1993). Adaptation canadienne-française de la forme révisée du State–Trait Anxiety Inventory de Spielberger [A French-Canadian adaptation of the revised version of Spielberger’s State–Trait Anxiety Inventory]. *Canadian Journal of Behavioural Science/Revue canadienne des sciences du comporteme*, *25*(4), 559–578.

[CIT0035] Gee, D. G., Humphreys, K. L., Flannery, J., Goff, B., Telzer, E. H., Shapiro, M., Hare, T. A., Bookheimer, S. Y., & Tottenham, N. (2013). A developmental shift from positive to negative connectivity in human amygdala-prefrontal circuitry. *The Journal of Neuroscience*, *33*(10), 4584–4593. 10.1523/JNEUROSCI.3446-12.2013 23467374 PMC3670947

[CIT0036] Giourou, E., Skokou, M., Andrew, S. P., Alexopoulou, K., Gourzis, P., & Jelastopulu, E. (2018). Complex posttraumatic stress disorder: The need to consolidate a distinct clinical syndrome or to reevaluate features of psychiatric disorders following interpersonal trauma? *World Journal of Psychiatry*, *8*(1), 12–19. 10.5498/wjp.v8.i1.12 29568727 PMC5862650

[CIT0037] Glover, E. M., Phifer, J. E., Crain, D. F., Norrholm, S. D., Davis, M., Bradley, B., Ressler, K. J., & Jovanovic, T. (2011). Tools for translational neuroscience: PTSD is associated with heightened fear responses using acoustic startle but not skin conductance measures. *Depression and Anxiety*, *28*(12), 1058–1066. 10.1002/da.20880 21898707 PMC3229665

[CIT0038] Gogolla, N., Caroni, P., Lüthi, A., & Herry, C. (2009). Perineuronal nets protect fear memories from erasure. *Science*, *325*(5945), 1258–1261. 10.1126/science.1174146 19729657

[CIT0039] Gray, M. J., Litz, B. T., Hsu, J. L., & Lombardo, T. W. (2004). Psychometric properties of the life events checklist. *Assessment*, *11*(4), 330–341. 15486169 10.1177/1073191104269954

[CIT0040] Haering, S., Schulze, L., Geiling, A., Meyer, C., Klusmann, H., Schumacher, S., Knaevelsrud, C., & Engel, S. (2024). Higher risk-less data: A systematic review and meta-analysis on the role of sex and gender in trauma research. *Journal of Psychopathology and Clinical Science*, *133*(3), 257–272. 10.1037/abn0000899 38619461

[CIT0041] Hanson, J. L., Chung, M. K., Avants, B. B., Rudolph, K. D., Shirtcliff, E. A., Gee, J. C., Davidson, R. J., & Pollak, S. D. (2012). Structural variations in prefrontal cortex mediate the relationship between early childhood stress and spatial working memory. *Journal of Neuroscience*, *32*(23), 7917–7925. 10.1523/JNEUROSCI.0307-12.2012 22674267 PMC3375595

[CIT0042] Helpman, L., Marin, M.-F., Papini, S., Zhu, X., Sullivan, G. M., Schneier, F., Neria, M., Shvil, E., Malaga Aragon, M. J., Markowitz, J. C., Lindquist, M. A., Wager, T. D., Milad, M. R., & Neria, Y. (2016). Neural changes in extinction recall following prolonged exposure treatment for PTSD: A longitudinal fMRI study. *NeuroImage: Clinical*, *12*, 715–723. 10.1016/j.nicl.2016.10.007 27761402 PMC5065048

[CIT0043] Hopper, J. W., Frewen, P. A., van der Kolk, B. A., & Lanius, R. A. (2007). Neural correlates of reexperiencing, avoidance, and dissociation in PTSD: Symptom dimensions and emotion dysregulation in responses to script-driven trauma imagery. *Journal of Traumatic Stress*, *20*(5), 713–725. 10.1002/jts.20284 17955540

[CIT0044] Jaworska, N., & MacQueen, G. (2015). Adolescence as a unique developmental period. *Journal of Psychiatry and Neuroscience*, *40*(5), 291–293. 10.1503/jpn.150268 26290063 PMC4543091

[CIT0045] Jovanovic, T., Kazama, A., Bachevalier, J., & Davis, M. (2012). Impaired safety signal learning may be a biomarker of PTSD. *Neuropharmacology*, *62*(2), 695–704. 10.1016/j.neuropharm.2011.02.023 21377482 PMC3146576

[CIT0046] Kessler, R. C. (1995). Posttraumatic stress disorder in the national comorbidity survey. *Archives of General Psychiatry*, *52*(12), 1048–1060. 10.1001/archpsyc.1995.03950240066012 7492257

[CIT0047] Kessler, R. C., Berglund, P., Demler, O., Jin, R., Merikangas, K. R., & Walters, E. E. (2005). Lifetime prevalence and age-of-onset distributions of DSM-IV disorders in the national comorbidity survey replication. *Archives of General Psychiatry*, *62*(6), 593–602. 10.1001/archpsyc.62.6.593 15939837

[CIT0048] Kilpatrick, D. G., Resnick, H. S., Milanak, M. E., Miller, M. W., Keyes, K. M., & Friedman, M. J. (2013). National estimates of exposure to traumatic events and PTSD prevalence using DSM-IV and DSM-5 criteria. *Journal of Traumatic Stress*, *26*(5), 537–547. 10.1002/jts.21848 24151000 PMC4096796

[CIT0049] Kimerling, R., Allen, M. C., & Duncan, L. E. (2018). Chromosomes to social contexts: Sex and gender differences in PTSD. *Current Psychiatry Reports*, *20*(12), 114. 10.1007/s11920-018-0981-0 30345456

[CIT0050] Kreutzmann, J. C., Marin, M.-F., Fendt, M., Milad, M. R., Ressler, K., & Jovanovic, T. (2021). Unconditioned response to an aversive stimulus as predictor of response to conditioned fear and safety: A cross-species study. *Behavioural Brain Research*, *402*, 113105. 10.1016/j.bbr.2020.113105 33417995

[CIT0051] Krystal, J. H., & Duman, R. (2004). What’s missing in posttraumatic stress disorder research? Studies of human postmortem tissue. *Psychiatry Interpers Biol Process*, *67*(4), 398–403. 10.1521/psyc.67.4.398.56567 15801380

[CIT0052] Kuznetsova, A., Brockhoff, P. B., & Christensen, R. H. B. (2017). Lmertest package: Tests in linear mixed effects models. *Journal of Statistical Software*, *82*(13), 1–26. 10.18637/jss.v082.i13

[CIT0053] Lebron-Milad, K., & Milad, M. R. (2012). Sex differences, gonadal hormones and the fear extinction network: Implications for anxiety disorders. *Biology of Mood & Anxiety Disorders*, *2*(1), 3. 10.1186/2045-5380-2-3 22738383 PMC3384233

[CIT0054] LeDoux, J. E. (2000). Emotion circuits in the brain. *Annual Review of Neuroscience*, *23*(1), 155–184. 10.1146/annurev.neuro.23.1.155 10845062

[CIT0055] Lenth, R. V., Piaskowski, J., Banfai, B., Bolker, B., Buerkner, P., Giné-Vázquez, I., Hervé, M., Jung, M., Love, J., Miguez, F., Riebl, H., & Singmann, H. (2025). emmeans: Estimated marginal means, aka least-squares means.

[CIT0056] Lilly, M. M., & Valdez, C. E. (2012). Interpersonal trauma and PTSD: The roles of gender and a lifespan perspective in predicting risk. *Psychological Trauma: Theory, Research, Practice, and Policy*, *4*(1), 140–144. 10.1037/a0022947

[CIT0057] Lonsdorf, T. B., Menz, M. M., Andreatta, M., Fullana, M. A., Golkar, A., Haaker, J., Heitland, I., Hermann, A., Kuhn, M., Kruse, O., Meir Drexler, S., Meulders, A., Nees, F., Pittig, A., Richter, J., Römer, S., Shiban, Y., Schmitz, A., Straube, B., … Merz, C. J. (2017). Don’t fear “fear conditioning”: Methodological considerations for the design and analysis of studies on human fear acquisition, extinction, and return of fear. *Neuroscience & Biobehavioral Reviews*, *77*, 247–285. 10.1016/j.neubiorev.2017.02.026 28263758

[CIT0058] Lonsdorf, T. B., & Merz, C. J. (2017). More than just noise: Inter-individual differences in fear acquisition, extinction and return of fear in humans – Biological, experiential, temperamental factors, and methodological pitfalls. *Neuroscience & Biobehavioral Reviews*, *80*, 703–728. 10.1016/j.neubiorev.2017.07.007 28764976

[CIT0059] Lupien, S. J., McEwen, B. S., Gunnar, M. R., & Heim, C. (2009). Effects of stress throughout the lifespan on the brain, behaviour and cognition. *Nature Reviews Neuroscience*, *10*(6), 434–445. 10.1038/nrn2639 19401723

[CIT0060] Marin, M.-F., Bilodeau-Houle, A., Morand-Beaulieu, S., Brouillard, A., Herringa, R. J., & Milad, M. R. (2020). Vicarious conditioned fear acquisition and extinction in child-parent dyads. *Scientific Reports*, *10*(1), 17130. 10.1038/s41598-020-74170-1 33051522 PMC7555483

[CIT0061] Marin, M.-F., Song, H., VanElzakker, M. B., Staples-Bradley, L. K., Linnman, C., Pace-Schott, E. F., Lasko, N. B., Shin, L. M., & Milad, M. R. (2016). Association of resting metabolism in the fear neural network with extinction recall activations and clinical measures in trauma-exposed individuals. *American Journal of Psychiatry*, *173*(9), 930–938. 10.1176/appi.ajp.2015.14111460 26917165 PMC13035175

[CIT0062] Matuschek, H., Kliegl, R., Vasishth, S., Baayen, H., & Bates, D. (2017). Balancing Type I error and power in linear mixed models. *Journal of Memory and Language*, *94*, 305–315. 10.1016/j.jml.2017.01.001

[CIT0063] McLaughlin, K. A., Sheridan, M. A., & Lambert, H. K. (2014). Childhood adversity and neural development: Deprivation and threat as distinct dimensions of early experience. *Neuroscience & Biobehavioral Reviews*, *47*, 578–591. 10.1016/j.neubiorev.2014.10.012 25454359 PMC4308474

[CIT0064] Milad, M. R., Orr, S. P., Lasko, N. B., Chang, Y., Rauch, S. L., & Pitman, R. K. (2008). Presence and acquired origin of reduced recall for fear extinction in PTSD: Results of a twin study. *Journal of Psychiatric Research*, *42*(7), 515–520. 10.1016/j.jpsychires.2008.01.017 18313695 PMC2377011

[CIT0065] Milad, M. R., Orr, S. P., Pitman, R. K., & Rauch, S. L. (2005). Context modulation of memory for fear extinction in humans. *Psychophysiology*, *42*(4), 456–464. 10.1111/j.1469-8986.2005.00302.x 16008774

[CIT0066] Milad, M. R., Pitman, R. K., Ellis, C. B., Gold, A. L., Shin, L. M., Lasko, N. B., Zeidan, M. A., Handwerger, K., Orr, S. P., & Rauch, S. L. (2009). Neurobiological basis of failure to recall extinction memory in posttraumatic stress disorder. *Biological Psychiatry*, *66*(12), 1075–1082. 10.1016/j.biopsych.2009.06.026 19748076 PMC2787650

[CIT0067] Milad, M. R., & Quirk, G. J. (2012). Fear extinction as a model for translational neuroscience: Ten years of progress. *Annual Review of Psychology*, *63*(1), 129–151. 10.1146/annurev.psych.121208.131631 PMC4942586 22129456

[CIT0068] Milad, M. R., Rauch, S. L., Pitman, R. K., & Quirk, G. J. (2006). Fear extinction in rats: Implications for human brain imaging and anxiety disorders. *Biological Psychology*, *73*(1), 61–71. 10.1016/j.biopsycho.2006.01.008 16476517

[CIT0069] Milad, M. R., Wright, C. I., Orr, S. P., Pitman, R. K., Quirk, G. J., & Rauch, S. L. (2007). Recall of fear extinction in humans activates the ventromedial prefrontal cortex and hippocampus in concert. *Biological Psychiatry*, *62*(5), 446–454. 10.1016/j.biopsych.2006.10.011 17217927

[CIT0070] Morina, N., Wicherts, J. M., Lobbrecht, J., & Priebe, S. (2014). Remission from post-traumatic stress disorder in adults: A systematic review and meta-analysis of long term outcome studies. *Clinical Psychology Review*, *34*(3), 249–255. 10.1016/j.cpr.2014.03.002 24681171

[CIT0071] Nelson, C. A., & Gabard-Durnam, L. J. (2020). Early adversity and critical periods: Neurodevelopmental consequences of violating the expectable environment. *Trends in Neurosciences*, *43*(3), 133–143. 10.1016/j.tins.2020.01.002 32101708 PMC8092448

[CIT0072] North, C. S., Oliver, J., & Pandya, A. (2012). Examining a comprehensive model of disaster-related posttraumatic stress disorder in systematically studied survivors of 10 disasters. *American Journal of Public Health*, *102*(10), e40–e48. 10.2105/AJPH.2012.300689 22897543 PMC3490647

[CIT0073] Olff, M., Langeland, W., Draijer, N., & Gersons, B. P. R. (2007). Gender differences in posttraumatic stress disorder. *Psychological Bulletin*, *133*(2), 183–204. 10.1037/0033-2909.133.2.183 17338596

[CIT0074] Ozer, E. J., Best, S. R., Lipsey, T. L., & Weiss, D. S. (2003). Predictors of posttraumatic stress disorder and symptoms in adults: A meta-analysis. *Psychological Bulletin*, *129*(1), 52–73. 10.1037/0033-2909.129.1.52 12555794

[CIT0075] Pechtel, P., & Pizzagalli, D. A. (2011). Effects of early life stress on cognitive and affective function: An integrated review of human literature. *Psychopharmacology*, *214*(1), 55–70. 10.1007/s00213-010-2009-2 20865251 PMC3050094

[CIT0076] Pineles, S. L., Arditte Hall, K. A., & Rasmusson, A. M. (2017). Gender and PTSD: Different pathways to a similar phenotype. *Current Opinion in Psychology*, *14*, 44–48. 10.1016/j.copsyc.2016.11.002 28813318

[CIT0077] Pitman, R. K., Rasmusson, A. M., Koenen, K. C., Shin, L. M., Orr, S. P., Gilbertson, M. W., Milad, M. R., & Liberzon, I. (2012). Biological studies of post-traumatic stress disorder. *Nature Reviews Neuroscience*, *13*(11), 769–787. 10.1038/nrn3339 23047775 PMC4951157

[CIT1001] R Core Team. (2023). *R: A language and environment for statistical computing*. R Foundation for Statistical Computing.

[CIT0078] Rougemont-Bücking, A., Linnman, C., Zeffiro, T. A., Zeidan, M. A., Lebron-Milad, K., Rodriguez-Romaguera, J., Rauch, S. L., Pitman, R. K., & Milad, M. R. (2011). Altered processing of contextual information during fear extinction in PTSD: An fMRI study. *CNS Neuroscience & Therapeutics*, *17*(4), 227–236. 10.1111/j.1755-5949.2010.00152.x 20406268 PMC6493793

[CIT0079] Rytwinski, N. K., Scur, M. D., Feeny, N. C., & Youngstrom, E. A. (2013). The co-occurrence of major depressive disorder among individuals with posttraumatic stress disorder: A meta-analysis. *Journal of Traumatic Stress*, *26*(3), 299–309. 10.1002/jts.21814 23696449

[CIT0080] Sheridan, M. A., & McLaughlin, K. A. (2014). Dimensions of early experience and neural development: Deprivation and threat. *Trends in Cognitive Sciences*, *18*(11), 580–585. 10.1016/j.tics.2014.09.001 25305194 PMC4252647

[CIT0081] Shin, L. M., & Liberzon, I. (2010). The neurocircuitry of fear, stress, and anxiety disorders. *Neuropsychopharmacology*, *35*(1), 169–191. 10.1038/npp.2009.83 19625997 PMC3055419

[CIT0082] Shin, L. M., Rauch, S. L., & Pitman, R. K. (2006). Amygdala, medial prefrontal cortex, and hippocampal function in PTSD. *Annals of the New York Academy of Sciences*, *1071*(1), 67–79. 10.1196/annals.1364.007 16891563

[CIT0083] Spielberger, C. D. (1983). *State-trait anxiety inventory for adults (STAI-AD)* [Database record]. APA PsycTests.

[CIT0084] Stevens, J. S., Jovanovic, T., Fani, N., Ely, T. D., Glover, E. M., Bradley, B., & Ressler, K. J. (2013). Disrupted amygdala-prefrontal functional connectivity in civilian women with posttraumatic stress disorder. *Journal of Psychiatric Research*, *47*(10), 1469–1478. 10.1016/j.jpsychires.2013.05.031 23827769 PMC3743923

[CIT0085] Stevens, J. S., van Rooij, S. J., & Jovanovic, T. (2018). Developmental contributors to trauma response: The importance of sensitive periods, early environment, and sex differences. *Current Topics in Behavioral Neurosciences*, *38*, 1–22. 27830573 10.1007/7854_2016_38 PMC5425320

[CIT0086] Stockhorst, U., & Antov, M. I. (2016). Modulation of fear extinction by stress, stress hormones and estradiol: A review. *Frontiers in Behavioral Neuroscience*, *9*, 359. 10.3389/fnbeh.2015.00359 26858616 PMC4726806

[CIT0087] Teicher, M. H., & Samson, J. A. (2016). Annual research review: Enduring neurobiological effects of childhood abuse and neglect. *Journal of Child Psychology and Psychiatry*, *57*(3), 241–266. 10.1111/jcpp.12507 26831814 PMC4760853

[CIT0088] Tolin, D. F., & Foa, E. B. (2008). Sex differences in trauma and posttraumatic stress disorder: A quantitative review of 25 years of research. *Psychological Trauma: Theory, Research, Practice, and Policy*, *S*(1), 37–85. 10.1037/1942-9681.S.1.37 17073529

[CIT0089] Tottenham, N., & Galván, A. (2016). Stress and the adolescent brain: Amygdala-prefrontal cortex circuitry and ventral striatum as developmental targets. *Neuroscience & Biobehavioral Reviews*, *70*, 217–227. 10.1016/j.neubiorev.2016.07.030 27473936 PMC5074883

[CIT0090] Tottenham, N., & Sheridan, M. A. (2010). A review of adversity, the amygdala and the hippocampus: A consideration of developmental timing. *Frontiers in Human Neuroscience*, *3*, 1019. 10.3389/neuro.09.068.2009 PMC2813726 20161700

[CIT0091] Uematsu, A., Matsui, M., Tanaka, C., Takahashi, T., Noguchi, K., Suzuki, M., & Nishijo, H. (2012). Developmental trajectories of amygdala and hippocampus from infancy to early adulthood in healthy individuals. *PLoS One*, *7*(10), e46970. 10.1371/journal.pone.0046970 23056545 PMC3467280

[CIT0092] Van Ameringen, M., Mancini, C., Patterson, B., & Boyle, M. H. (2008). Post-traumatic stress disorder in Canada. *CNS Neuroscience & Therapeutics*, *14*(3), 171–181. 10.1111/j.1755-5949.2008.00049.x 18801110 PMC6494052

[CIT0093] van der Kolk, B. A., Pelcovitz, D., Roth, S., Mandel, F. S., McFarlane, A., & Herman, J. L. (1996). Dissociation, somatization, and affect dysregulation: The complexity of adaptation of trauma. *American Journal of Psychiatry*, *153*(7 Suppl), 83–93. 10.1176/ajp.153.7.83 8659645

[CIT0094] van Minnen, A., Zoellner, L. A., Harned, M. S., & Mills, K. (2015). Changes in comorbid conditions after prolonged exposure for PTSD: A literature review. *Current Psychiatry Reports*, *17*(3), 17. 10.1007/s11920-015-0549-1 25736701 PMC4348535

[CIT0095] Wang, Y.-P., & Gorenstein, C. (2013). Psychometric properties of the beck depression inventory-II: A comprehensive review. *Braz J Psychiatry*, *35*(4), 416–431. 10.1590/1516-4446-2012-1048 24402217

[CIT0096] Weathers, F. W., Litz, B. T., Keane, T. M., Palmieri, P. A., Marx, B. P., & Schnurr, P. P. (2013). The PTSD checklist for DSM-5 (PCL-5).

[CIT0097] Wicking, M., Steiger, F., Nees, F., Diener, S. J., Grimm, O., Ruttorf, M., Schad, L. R., Winkelmann, T., Wirtz, G., & Flor, H. (2016). Deficient fear extinction memory in posttraumatic stress disorder. *Neurobiology of Learning and Memory*, *136*, 116–126. 10.1016/j.nlm.2016.09.016 27686278

[CIT0098] Zlotnick, C., Johnson, J., Kohn, R., Vicente, B., Rioseco, P., & Saldivia, S. (2008). Childhood trauma, trauma in adulthood, and psychiatric diagnoses: Results from a community sample. *Comprehensive Psychiatry*, *49*(2), 163–169. 10.1016/j.comppsych.2007.08.007 18243889 PMC2648973

