# Supplementary material for: The role of the first interpersonal trauma exposure’s developmental period on fear regulation processes among adult women
Source: Eur J Psychotraumatol. 2025 Nov 19;16(1):2587483. doi: 10.1080/20008066.2025.2587483 (PMC12632224; doi:10.1080/20008066.2025.2587483)
Supplement: Supplementary_Material.docx [file ZEPT_A_2587483_SM4531.docx]

**Supplementary material**

**Figure S1.**

*General procedure*

The experiment took place over three sessions. On Day 1, participants provided written informed consent and completed the LEC-5 and PCL-5 questionnaires. On Day 2, which was scheduled between two to four weeks following Day 1, participants underwent the fear conditioning and extinction phases of the protocol. On Day 3, which occurred 24 hours after Day 2, participants completed a demographic questionnaire before taking part in the extinction memory recall phase. Afterwards, they filled out the BDI-II and STAI-T questionnaires. Finally, they were debriefed about the study and compensated for their participation.


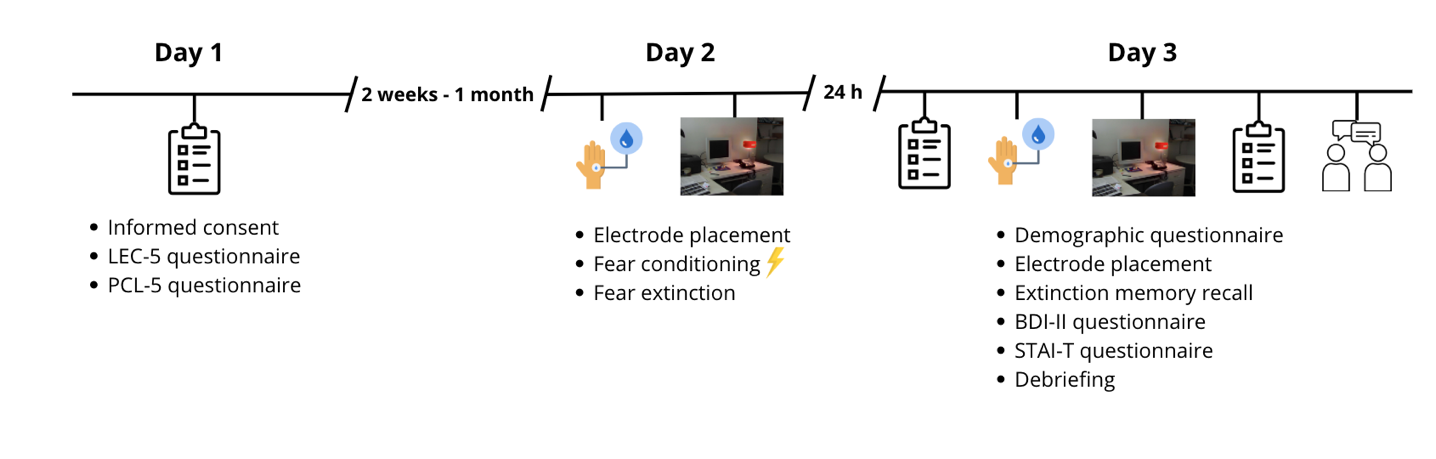


**Table S1**

*Effects for Conditioning, Extinction, and Extinction Recall Models with the Covariates*

| Effect | *F*(df_1_, df_2_) | *p* |
| --- | --- | --- |
| *Conditioning* |  |  |
| Interpersonal Trauma Subtypes | *F*(1, 53.92) = 0.02 | .88 |
| Trauma Recency Score | *F*(35, 53.90) = 0.78 | .78 |
| Trauma Age Group | *F*(2, 589.28) = 0.73 | .48 |
| Stimulus Type | *F*(1, 620.02) = 0.98 | .32 |
| Time | *F*(3, 620.07) = 31.83 | <.001 |
| Trauma Age Group x Stimulus Type | *F*(2, 620.01) = 0.49 | .61 |
| Trauma Age Group x Time | *F*(6, 620.06) = 0.76 | .60 |
| Stimulus Type x Time | *F*(3, 620.03) = 4.55 | 0.004 |
| Trauma Age Group x Stimulus Type x Time | *F*(6, 620.02) = 0.25 | .96 |
| *Extinction* |  |  |
| Interpersonal Trauma Subtypes | *F*(1, 53.97) = 2.36 | .13 |
| Trauma Recency Score | *F*(35, 53.94) = 1.11 | .36 |
| Trauma Age Group | *F*(2, 53.97) = 1.66 | .20 |
| Stimulus Type | *F*(1, 2093.02) = 50.37 | < .001 |
| Time | *F*(3, 2093.18) = 228.09 | < .001 |
| Trauma Age Group x Stimulus Type | *F*(2, 2093.01) = 2.85 | .06 |
| Trauma Age Group x Time | *F*(6, 2093.16) = 2.96 | .007 |
| Stimulus Type x Time | *F*(3, 2093.90) = 8.59 | < .001 |
| Trauma Age Group x Stimulus Type x Time | *F*(6, 2093.00) = 0.94 | .46 |
| *Extinction memory recall* |  |  |
| Interpersonal Trauma Subtypes | *F*(1, 51.98) = 0.99 | .32 |
| Trauma Recency Score | *F*(35, 51.98) = 1.03 | .46 |
| Trauma Age Group | *F*(2, 51.97) = 3.21 | .05 |
| Stimulus Type | *F*(1, 2055.02) = 30.92 | < .001 |
| Time | *F*(3, 2055.05) = 261.91 | < .001 |
| Trauma Age Group x Stimulus Type | *F*(2, 2055.02) = 2.25 | .11 |
| Trauma Age Group x Time | *F*(6, 2055.06) = 7.56 | < .001 |
| Stimulus Type x Time | *F*(3, 2055.03) = 13.41 | < .001 |
| Trauma Age Group x Stimulus Type x Time | *F*(6, 2055.02) = 1.42 | .20 |

**Note.** *F* = F-ratio; df = degrees of freedom.
